# Analysis of Hepatitis C Virus Genotype 1b Resistance Variants in Japanese Patients Treated with Paritaprevir-Ritonavir and Ombitasvir

**DOI:** 10.1128/AAC.02606-15

**Published:** 2016-01-29

**Authors:** Preethi Krishnan, Gretja Schnell, Rakesh Tripathi, Jill Beyer, Thomas Reisch, Xinyan Zhang, Carolyn Setze, Lino Rodrigues, Margaret Burroughs, Rebecca Redman, Kazuaki Chayama, Hiromitsu Kumada, Christine Collins, Tami Pilot-Matias

**Affiliations:** a Research and Development, AbbVie Inc., North Chicago, Illinois, USA; b Department of Gastroenterology and Metabolism, Hiroshima University, Hiroshima, Japan; c Department of Hepatology, Toranomon Hospital, Tokyo, Japan

## Abstract

Treatment of HCV genotype 1b (GT1b)-infected Japanese patients with paritaprevir (NS3/4A inhibitor boosted with ritonavir) and ombitasvir (NS5A inhibitor) in studies M12-536 and GIFT-I demonstrated high sustained virologic response (SVR) rates. The virologic failure rate was 3% (13/436) across the two studies. Analyses were conducted to evaluate the impact of baseline resistance-associated variants (RAVs) on treatment outcome and the emergence and persistence of RAVs in patients experiencing virologic failure. Baseline paritaprevir resistance-conferring variants in NS3 were infrequent, while Y93H in NS5A was the most prevalent ombitasvir resistance-conferring variant at baseline. A comparison of baseline prevalence of polymorphisms in Japanese and western patients showed that Q80L and S122G in NS3 and L28M, R30Q, and Y93H in NS5A were significantly more prevalent in Japanese patients. In the GIFT-I study, the prevalence of Y93H in NS5A varied between 13% and 21% depending on the deep-sequencing detection threshold. Among patients with Y93H comprising <1%, 1 to 40%, or >40% of their preexisting viral population, the 24-week SVR (SVR_24_) rates were >99% (276/277), 93% (38/41), and 76% (25/33), respectively, indicating that the prevalence of Y93H within a patient's viral population is a good predictor of treatment response. The predominant RAVs at the time of virologic failure were D168A/V in NS3 and Y93H alone or in combination with other variants in NS5A. While levels of NS3 RAVs declined over time, NS5A RAVs persisted through posttreatment week 48. Results from these analyses are informative in understanding the resistance profile of an ombitasvir- plus paritaprevir/ritonavir-based regimen in Japanese GT1b-infected patients.

## INTRODUCTION

Hepatitis C virus (HCV) is an enveloped, single-stranded, positive-sense RNA virus in the Flaviviridae family that infects approximately 170 million people worldwide (1, 2). It is estimated that 2 million people in Japan are infected with HCV (3). Globally, 7 distinct HCV genotypes (GT) and 67 subtypes have been characterized (4). In Japan, approximately 70% of HCV infections are GT1b, and 25 to 30% are GT2a or GT2b (3). In contrast to the United States and many parts of Europe, in Japan very few HCV-infected patients (<1% of GT1-infected patients) are infected with GT1a (5).

Epidemiological and phylogenetic studies suggest that HCV began to infect large numbers of Japanese in the 1920s, southern Europeans in the 1940s, and North Americans in the 1960s and 1970s (6). Longitudinal studies have indicated that 1.44 × 10^−3^ nucleotide changes occur per site per year over the whole HCV genome (7, 8). This rapid sequence drift has led to the formation of separate strains or isolates with up to 10% nucleotide sequence variability within HCV subtypes (9). The prevalence of sequence polymorphisms within the same HCV subtype may differ across geographic regions depending on the timing and spread of the original infection (10). Such sequence differences may impact treatment outcomes with direct-acting antiviral regimens. Understanding the impact of preexisting polymorphisms on treatment outcome and identification of treatment-emergent resistance-associated variants (RAVs) in patients failing treatment with direct-acting antiviral therapy is important for the assessment of initial treatment and retreatment options.

Paritaprevir (NS3/4A protease inhibitor identified by AbbVie and Enanta and boosted with ritonavir; termed paritaprevir/r) and ombitasvir (NS5A inhibitor) have potent *in vitro* antiviral activity against multiple HCV genotypes, including 1a, 1b, 2a, 2b, 3a, 4a, 4d, and 6a (11, 12). The efficacy and safety of this interferon (IFN)- and ribavirin (RBV)-free 2-direct-acting-antiviral (2D) regimen has been evaluated in the phase 2 study M12-536 and the phase 3 study GIFT-I in Japan (13, 14). Both studies have demonstrated high sustained virological response (SVR) rates in treatment-naive and -experienced GT1b-infected patients (M12-536, 88.9% to 100%; GIFT-I, 90.5% to 98.1%) (13, 14).

Comparable efficacy results have been reported with ledipasvir plus sofosbuvir (15) and daclatasvir plus asunaprevir (16) regimens in Japanese HCV GT1b-infected patients. However, with the daclastavir-plus-asunaprevir regimen, the presence of NS5A variant L31M or Y93H at baseline (detected by population sequencing at a prevalence of 4% or 14%, respectively) was associated with an SVR rate of 25% or 43%, respectively (17, 18). NS5A variants L31M and Y93H also confer high levels of resistance to ledipasvir. In a Japanese phase 3 clinical trial, the 12-week SVR (SVR_12_) rates remained high with ledipasvir-plus-sofosbuvir-based regimens in patients with baseline variants in NS5A (15, 17). However, in phase 3 western studies with ledipasvir-plus-sofosbuvir-based regimens, the presence of NS5A variants conferring >100-fold resistance at baseline was associated with a lower SVR rate in treatment-experienced but not treatment-naive patients (19). Understanding the impact of baseline RAVs on treatment outcome is important, especially for compounds targeting HCV NS5A.

The objective of these analyses was to provide a comprehensive evaluation of viral resistance in HCV GT1b-infected patients in the Japanese studies M12-536 and GIFT-I. The impact of baseline variants on treatment outcome was evaluated, and the presence of treatment-emergent RAVs in the patients who experienced virologic failure in the two studies was assessed. In addition, the prevalence of baseline polymorphisms at resistance-associated amino acid positions in NS3 and NS5A was compared between Japanese and western patients.

## MATERIALS AND METHODS

### Patients and study design.

The phase 2 and 3 study designs, randomization procedures, and efficacy and safety analyses were previously described (13, 14). Briefly, in M12-536 (ClinicalTrials.gov identifier NCT01672983), 73 noncirrhotic HCV GT1b-infected patients were randomized in a 1:1:1:1 ratio to receive once-daily (QD) ombitasvir at 25 mg plus paritaprevir/r at 100/100 mg or 150/100 mg for 12 or 24 weeks. In the phase 3 GIFT-I study (ClinicalTrials.gov identifier NCT02023099), HCV GT1b-infected patients received a once-daily fixed-dose combination of ombitasvir/paritaprevir/r (25 mg/150 mg/100 mg) (termed the 2D regimen) for 12 weeks. Noncirrhotic patients were randomized in a 2:1 ratio to receive a double-blind 2D regimen (arm A; *n* = 215) or double-blind placebo (arm B; *n* = 106), and patients with compensated cirrhosis enrolled in arm C (*n* = 42) received open-label 2D. Patients receiving placebo in arm B subsequently received an open-label 2D regimen for 12 weeks. At the time of these analyses, all patients in arms A, B, and C had reached posttreatment week 24 or prematurely discontinued the study.

Available GT1b samples, predominantly those obtained from patients in the United States and Europe enrolled in the clinical studies AVIATOR, SAPPHIRE-I, SAPPHIRE-II, PEARL-I, PEARL-II, PEARL-III, and TURQUOISE-II, were utilized for baseline sequence analyses of the western patient population (20–27).

All patients provided written informed consent before enrolling in the studies. The studies were performed in accordance with good clinical practice guidelines and the principles of the Declaration of Helsinki, and the study protocols were approved by the relevant institutional review boards and regulatory agencies.

### Sample processing.

The GT1b-specific primers for reverse transcription-PCR (RT-PCR), nested PCR, and sequencing were designed based on the alignments of GT1b sequences in the European HCV database (28) in conserved regions flanking the gene of interest, with nucleotide degeneracies incorporated at positions where significant variability existed among the HCV sequences for the subtype. HCV RNA was purified from 550 μl of each plasma sample using an Abbott m2000 instrument (Abbott Molecular, Des Plaines, IL) and eluted in a final volume of 70 μl. The target genes, NS3/4A and NS5A, were amplified from 20 μl of HCV RNA by RT-PCR using the Superscript III one-step RT-PCR system with platinum *Taq* high fidelity (Invitrogen, Carlsbad, CA) followed by nested PCR using primers appropriate for GT1b sequences. Only samples with an HCV RNA level of ≥1,000 IU/ml were amplified in order to reduce the chance of oversampling bias. For patients who experienced virologic failure, the sample closest in time after virologic failure was utilized. For samples with an HCV RNA level of ≤50,000 IU/ml, RT-PCR was carried out in triplicate and the products were pooled prior to their use as a template for nested PCR. Population and (in some cases) clonal sequencing of NS3/4A and NS5A was conducted on the nested PCR products using gene-specific primers (25). At least two sequencing reads were performed in each direction across each target, providing a minimum of four sequencing reads.

### Sequence analyses.

Analyses for (i) prevalence of polymorphisms in Japanese and western GT1b sequences at resistance-associated amino acid positions in NS3 and NS5A by population sequencing, and a comparison using Fisher's exact test, and for (ii) analysis of treatment-emergent RAVs by population or clonal sequencing were performed using SAS, version 9.3 (SAS Institute, Inc., Cary, NC), under the UNIX operating system. Based on *in vitro* studies with HCV subgenomic replicons and phase 2 clinical studies of western HCV-infected patients, the following were identified as signature resistance-associated amino acid positions in HCV GT1b in baseline sequence analysis: 56, 155, 156, and 168 in NS3 for paritaprevir and 28, 29, 30, 31, 32, 58, and 93 in NS5A for ombitasvir (11, 12). Although variants at amino acid residues 54, 55, 80, and 122 in NS3 or 54, 62, and 92 in NS5A previously had not been associated with resistance to paritaprevir or ombitasvir in GT1b, these positions were included in the baseline sequence analysis due to the potential impact of variants at these positions on other NS3 protease or NS5A inhibitors (29, 30). Variants were identified based on comparison of baseline sequences to the reference sequence 1b-Con1 (GenBank accession number AJ238799). RAVs by clonal sequencing were defined as variants observed in 2 or more clones (out of at least 80 clones) from a sample obtained at a postbaseline time point relative to the reference sequence. Amino acid variants present prior to treatment in NS3 or NS5A that are not known to confer resistance to any inhibitor are referred to as baseline polymorphisms. Amino acid variants present at the baseline that are known to be associated with resistance to at least one member of the protease inhibitor or NS5A inhibitor class are referred to as baseline variants. Variants known to confer resistance to paritaprevir or ombitasvir are referred to as RAVs.

### Deep-sequencing analysis.

The deep sequencing of baseline samples from the GIFT-I study was conducted by DDL Diagnostic Laboratory (Rijswijk, Netherlands). PCR amplicons from baseline samples were purified using Ampure XP beads (Beckman Coulter Genomics) and quantified using the Quant-iT PicoGreen double-stranded DNA (dsDNA) kit (Life Technologies). The DNA then was fragmented and tagged using the Nextera XT sample preparation kit (Illumina, San Diego, CA) according to the manufacturer's instructions. Index primers were added by limited-cycle PCR using the Nextera XT Index kit (Illumina, San Diego, CA), and samples were normalized using beads with maximum binding capacity according to the Nextera XT sample preparation kit instructions. Multiplexed paired-end sequencing was conducted on the Illumina MiSeq platform using an MiSeq v2 sequencing kit with 300 cycles (Illumina). Demultiplexed FASTQ files then were mapped against the HCV 1b-Con1 (GenBank accession number AJ238799) reference sequence using CLC Genomics Workbench software (CLCBio, Denmark). Sequences were trimmed to remove nucleotides with a quality score (*Q*) lower than 30. An average of 94.3% of the reads had a *Q* of ≥30, and the average read length was 140 nucleotides. The minimum coverage was 5,000 sequencing reads. An amino acid variant report relative to the prototypic GT1b-Con1 reference sequence was generated with the Athena pipeline proprietary software (DDL Diagnostic Laboratory). The threshold for detection of amino acid polymorphisms by deep sequencing was set at 1%.

### Antiviral activity against a panel of NS3 or NS5A variants.

The methods describing the measurement of the effects of individual amino acid variants on the activity of an inhibitor in HCV replicon cell culture assays were described previously (11, 12). NS3 and NS5A variants each were introduced into the GT1b-Con1 subgenomic replicon plasmid using the Change-IT multiple-mutation site-directed mutagenesis kit (Affymetrix, Santa Clara, CA). In a transient assay, the replicon RNA containing the variant was transfected via electroporation into an Huh-7 cell line (31, 32). The luciferase activity in the cells was measured using a Victor II luminometer (Perkin-Elmer, Waltham, MA). The 50% effective concentrations (EC_50_s) of paritaprevir and ombitasvir, which were synthesized at AbbVie (33), were calculated using a nonlinear regression curve fit to the 4-parameter logistic equation in GraphPad Prism 4 software.

## RESULTS

### Analysis of polymorphisms in NS3/4A and NS5A at baseline by population sequencing.

The baseline amino acid variants detected by population sequencing at positions associated with resistance to NS3/4A or NS5A inhibitors in GT1b sequences from Japanese and western patients are shown in Table 1.

**TABLE 1:** Prevalence of baseline polymorphisms in NS3 and NS5A in GT1b-infected patients by population sequencing

Target and baseline polymorphism	Prevalence (%; *n*/*N*)^*a*^ in:	
	Japanese patients	Western patients
NS3		
T54S	3.3 (14/424)	1.6 (6/371)
V55A/I	0.2 (1/424)	1.0 (4/371)
Y56F	36.1 (153/424)	33.4 (124/371)
Q80H/I/K/M/R	2.1 (9/424)	0.3 (1/371)
Q80L	10.6 (45/424)	5.1 (19/371)
S122A/C/D/I/N/R/T/V/Y	10.9 (46/424)	10.8 (40/371)
S122G	26.2 (111/424)	5.1 (19/371)
A156T/V		0.5 (2/371)
D168E	1.2 (5/424)	0.3 (1/371)
NS5A		
L28I/V	0.7 (3/431)	
L28 M	8.8 (38/431)	1.3 (5/391)
R30G/H/L	0.7 (3/431)	0.5 (2/391)
R30Q	12.8 (55/431)	7.2 (28/391)
L31F/I/M	2.8 (12/431)	5.1 (20/391)
Q54A/C/D/E/H/K/L/N/P/R/S/V/Y	43.9 (189/431)	45.3 (177/391)
P58A/L/Q/R/S/T	7.4 (32/431)	7.4 (29/391)
Q62A/C/D/E/H/K/L/M/N/P/R/S/Y	9.7 (42/431)	10.0 (39/391)
A92E/K/M/S/T/V	7.4 (32/431)	4.9 (19/391)
Y93C/H/S	12.5 (54/431)	7.7 (30/391)

a Data are percentages of subjects with variants at the corresponding amino acid position. *n,* number of subjects with baseline variant; N, total number of samples sequenced.

Polymorphisms in NS3 at one of the amino acid positions 54, 55, 56, 80, 122, 155, 156, and 168 were detected in 62.0% (263/424) and 48.8% (181/371) of the Japanese and western patients, respectively. Variants conferring resistance to paritaprevir at amino acid position 155, 156, or 168 were rare (1.2%) in both populations. Polymorphisms at amino acid positions 56, 80, and 122 in NS3 were highly prevalent. While Y56F was observed at similar frequencies across both populations, polymorphisms at amino acid positions 80 and 122 were approximately 2-fold more prevalent in Japanese patients. Q80L and S122G were the predominant polymorphisms at the respective amino acid positions, and both were detected at a significantly higher prevalence in Japanese patients than in western patients (Q80L, *P* = 0.008; S122G, *P* < 0.001).

Polymorphisms in NS5A at one of the amino acid positions 28, 29, 30, 31, 32, 54, 58, 62, and 93 were detected in 67.1% (289/431) and 60.9% (238/391) of the Japanese and western patients, respectively. Polymorphisms at amino acid positions 28 and 30 were detected at a higher prevalence in Japanese patients, while the prevalence of polymorphisms at amino acid positions 31, 54, 58, 62, and 92 was similar across both populations. Q54H, P58S, Q62E, and A92T were the predominant polymorphisms at their respective amino acid positions in both populations. L28M and R30Q were the predominant variants at the respective amino acid positions, and both were detected at a significantly higher prevalence in Japanese patients than in western patients (L28M, *P* < 0.001; R30Q, *P* = 0.008). Y93H, which confers 77-fold resistance to ombitasvir (12), was observed in Japanese patients at a higher prevalence than in western patients (12.3% versus 7.4%; *P* = 0.020).

None of the Japanese or western patients had resistance-conferring baseline variants in both NS3 and NS5A by population sequencing.

### Impact of baseline variants on SVR_24_ in Japanese patients.

The impact of baseline variants on treatment outcome in studies M12-536 and GIFT-I was evaluated by comparing the 24-week SVR rates (SVR_24_) in patients with baseline variants at each amino acid position (54, 55, 56, 80, 122, or 168 in NS3 and 28, 30, 31, 54, 58, 62, 92, or 93 in NS5A) to the SVR_24_ rates in patients with the wild-type amino acid at the corresponding position.

Baseline sequence analysis in M12-536 utilized data from population sequencing. The SVR_24_ rates in patients with variants at baseline in NS3 or NS5A were similar to SVR_24_ rates in patients with the wild-type amino acid at each of the corresponding positions (Table 2). Four patients had Y93H at baseline without any additional variants at resistance-associated amino acid positions within NS5A, and all achieved SVR_24_.

**TABLE 2:** Impact of baseline variants in NS3 and NS5A by population sequencing on treatment outcome in M12-536 noncirrhotic patients

Target and baseline variant	SVR_24_ rate (%; *n*/*N*) with^*a*^:	
	Variant	Wild type
NS3		
Y56F	96 (24/25)	100 (45/45)
Q80H/I/K/L/M	88 (7/8)	100 (62/62)
S122G/N/T	100 (19/19)	98 (50/51)
NS5A		
L28 M	80 (4/5)	100 (66/66)
R30Q	88 (7/8)	100 (63/63)
L31 M	100 (3/3)	99 (67/68)
Q54H/L/N/Y	100 (36/36)	97 (34/35)
P58A/L/Q/S/T	100 (7/7)	98 (63/64)
Q62A/E	100 (5/5)	98 (65/66)
A92T	100 (5/5)	98 (65/66)
Y93H	100 (4/4)	99 (66/67)

a Patients not achieving SVR due to nonvirologic reasons, e.g., early discontinuations, missing SVR time point, etc., are excluded from the analysis. Only patients with available sequences (*N*) are included in the analysis. Therefore, N is less than the number of patients enrolled in the study and differs by target.

Baseline sequence analysis in GIFT-I (noncirrhotic and cirrhotic patient populations) utilized both population and deep-sequencing data (Tables 3 and 4). The threshold for the detection of amino acid variants by deep sequencing was set at 1%. Comparison of prevalence by population and deep sequencing indicated that the detection limit by population sequencing was approximately 15%. The impact of baseline variants on treatment outcome by deep sequencing was evaluated based on the prevalence of variants (1 to 15% or >15% for all variants; 1 to <5%, 5 to <15%, 15 to <40%, and >40% for Y93H) within a patient's viral population at each amino acid position.

**TABLE 3:** Impact of baseline variants on treatment outcome in GIFT-I

Target and variant	SVR_24_ rate (%; *n*/*N*)^*a*^ for:							
	Noncirrhotic patients				Cirrhotic patients			
	Detection threshold by deep sequencing			Variant detected by population sequencing	Detection threshold by deep sequencing			Variant detected by population sequencing
	1 to 15%	>15%	<1% (wild-type)		1 to 15%	>15%	<1% (wild-type)	
NS3								
T54S		100 (12/12)	97 (294/303)	100 (13/13)		100 (1/1)	92 (36/39)	100 (1/1)
V55I		100 (1/1)	97 (305/314)	100 (1/1)				
Y56F	100 (3/3)	97 (115/118)	97 (188/194)	97 (113/116)	100 (3/3)	92 (11/12)	92 (23/25)	91 (10/11)
Q80 H/K/L/M/N/R	89 (8/9)	95 (38/40)	98 (260/266)	95 (37/39)	100 (4/4)	100 (5/5)	90 (28/31)	100 (4/4)
S122 A/C/G/N/T/V	98 (40/41)	98 (114/116)	96 (152/158)	97 (112/116)	100 (6/6)	84 (16/19)	100 (15/15)	83 (15/18)
D168E	100 (4/4)	100 (3/3)	97 (299/308)	100 (3/3)		100 (1/1)	92 (36/39)	100 (1/1)
NS5A								
L28I/M/V/R	100 (4/4)	97 (30/31)	97 (268/276)	97 (30/31)		100 (4/4)	92 (33/36)	100 (4/4)
R30K/L/Q/R	100 (7/7)	98 (42/43)	97 (253/261)	98 (43/44)	100 (1/1)	100 (4/4)	91 (32/35)	100 (4/4)
L31I/F/M/V	100 (5/5)	100 (7/7)	97 (290/299)	100 (5/5)		50 (1/2)	95 (36/38)	50 (1/2)
Q54A/C/E/H/N/L/S/T/V/Y	94 (30/32)	98 (124/127)	97 (148/152)	98 (125/128)	100 (4/4)	95 (20/21)	87 (13/15)	95 (20/21)
P58A/L/Q/S/T/R	85 (11/13)	100 (22/22)	97 (269/276)	100 (23/23)	100 (2/2)	100 (2/2)	99 (33/36)	100 (2/2)
Q62A/C/D/E/H/K/M/N/P/L/S/R	92 (11/12)	100 (32/32)	97 (259/267)	97 (32/33)		100 (2/2)	92 (35/38)	100 (2/2)
A92E/S/T/V	100 (14/14)	94 (17/18)	97 (271/279)	95 (20/21)	67 (2/3)	100 (4/4)	94 (31/33)	80 (4/5)
Y93F	100 (1/1)							
Y93S		100 (1/1)		100 (1/1)				

a Patients not achieving SVR due to nonvirologic reasons, e.g., early discontinuations, missing SVR time point, etc., are excluded from the analysis. Only patients with available sequences (*N*) are included in the analysis. Therefore, N is less than the number of patients enrolled in the study and differs by target.

**TABLE 4:** Impact of Y93H in NS5A at baseline on treatment outcome in GIFT-I

Y93H type	SVR_24_ rate (%; *n*/*N*)^*a*^					
	Detection threshold by deep sequencing					Detection by population sequencing
	1 to <5%	5 to <15%	15 to 40%	>40%	<1% (wild type)	
Noncirrhotic	94 (16/17)	100 (8/8)	91 (10/11)	78 (21/27)	100^*b*^ (247/248)	82 (32/39)
Cirrhotic	100 (3/3)	0 (0/1)	100 (1/1)	67 (4/6)	100 (29/29)	71 (5/7)

a Patients not achieving SVR due to nonvirologic reasons, e.g., early discontinuations, missing SVR time point, etc., are excluded from the analysis. Only patients with available sequences (*N*) are included in the analysis. Therefore, N is less than the number of patients enrolled in the study.

b Includes 2 patients with Y93F or Y93S at baseline.

Among both noncirrhotic and cirrhotic patients in GIFT-I, SVR_24_ rates in the presence or absence of NS3 variants were similar. All patients with D168E at baseline achieved SVR_24_.

NS5A variants at amino acid position 28, 30, 54, 58, 62, or 92 also had no impact on treatment outcome in the GIFT-I study. Four patients with L31F and 1 patient each with Y93F or Y93S all achieved SVR_24_. The patient not achieving SVR_24_ with L31M at baseline also had a preexisting Y93H variant.

In GIFT-I, the prevalence of Y93H in NS5A at baseline by deep sequencing was 13% at a detection threshold of 15% and 21% at a detection threshold of 1%. In this study, the SVR_24_ rate in patients with detectable Y93H at baseline (≥1% by deep sequencing) was 85% (63/74), compared with >99% (276/277) in patients without detectable Y93H. In noncirrhotic patients, when Y93H was detected at a prevalence of ≤40% versus >40% within a patient's viral population, the SVR_24_ rates were 94% (34/36) and 78% (21/27), respectively. In the cirrhotic patients, the SVR_24_ rates were 80% (4/5) and 67% (4/6) when Y93H was present at a prevalence of ≤40% versus >40%, respectively. Therefore, Y93H at a prevalence of >40% within a patient's viral population appears to have the highest impact on response, with 76% (25/33) of the patients in this subset achieving SVR_24_.

Of the patients who had Y93H at baseline in M12-536 by population sequencing and in GIFT-I at a prevalence of >15% (equivalent to the detection limit by population sequencing), 59% (29/49) also had one or more additional variants at amino acid positions 28, 30, 31, 54, 58, 62, and/or 92 at a prevalence of >15%. The presence of multiple variants at baseline had no additional impact on treatment outcome, as the SVR_24_ rate in patients with Y93H alone was 80% (16/20), whereas it was 83% (24/29) in patients with multiple variants (Table 5).

**TABLE 5:** Impact of baseline Y93H alone or in combination with other variants in NS5A on treatment outcome

Variant(s)^*a*^	SVR_24_ rate (%; *n/N*)^*b*^ by study group			
	M12-536	GIFT-I		Total
		Noncirrhotic	Cirrhotic	
Y93H	100 (4/4)	73 (8/11)	80 (4/5)	80 (16/20)
R30Q, Y93H		100 (1/1)		
L31M, Y93H			(0/1)	
Q54C/H/L/R/Y, Y93H		91 (10/11)	100 (1/1)	
P58L/S, Y93H		100 (2/2)		
Q62D/H, Y93H		100 (2/2)		
A92V, Y93H		100 (1/1)		
L28M, R30Q, Y93H		(0/1)		
R30Q, Q54H/Y, Y93H		100 (1/1)		
Q54H, P58Q, Y93H		100 (1/1)		
Q54H/Y, Q62A/E/L, Y93H		67 (2/3)		
Q54H/Y, A92E/M/T/V, Y93H		50 (1/2)		
L28M, R30Q, P58S, Q62H, Y93H		100 (1/1)		
L28M, R30L, Q54H, P58L, A92V, Y93H		100 (1/1)		
Y93H/S in combination with other variants				83 (24/29)

a List includes variants detected from patient isolates; variants may not all be linked. Variants detected by population sequencing in study M12-536 and those detected by deep sequencing (detection threshold of >15%) in the GIFT-I study are reported.

b Patients not achieving SVR due to nonvirologic reasons, e.g., early discontinuations, missing SVR time point, etc., are excluded from the analysis. Only patients with available sequences (*N*) are included in the analysis. Therefore, N is less than the number of patients enrolled in the study and differs by target.

### Treatment-emergent RAVs in Japanese patients experiencing virologic failure.

Of the 73 GT1b-infected patients in M12-536, 1 patient receiving ombitasvir and the higher dose of 150/100 mg of paritaprevir/r for 12 weeks experienced virologic failure. In GIFT-I, 12 GT1b-infected patients experienced virologic failure, 9 out of 321 noncirrhotic patients and 3 out of 42 patients with cirrhosis. One of the 9 noncirrhotic patients experienced virologic failure in the posttreatment week 24 window. RAVs detected in the 13 patients at baseline, time of failure, and follow-up time points are shown in Table 6, and the activity of paritaprevir or ombitasvir against these RAVs in the GT1b-Con1 replicon is shown in Table 7.

**TABLE 6:** RAVs in NS3 and NS5A in patients who experienced virologic failure in M12-536 and GIFT-I^*i*^

Study arm^*a*^, VF type, time point	RAVs at each time point							
	NS3				NS5A			
	Baseline	Time of VF	PTW24	PTW48	Baseline	Time of VF	PTW24	PTW48
M12-536								
2, relapse, PTW2^*b*^^,^^*d*^	None^*f*^	D168V	None^*g*^	None	L28M + R30Q	L28M + R30Q + Y93H	L28M + R30Q + Y93H	L28M + R30Q, Y93H/Y
GIFT-I								
A, breakthrough, W6^*b*^^,^^*d*^	None^*h*^	Y56H + D168V	None^*g*^	None	Y93H^*h*^	Y93H	Y93H	Y93H
A, relapse, PTW2*^c,e^*	None^*h*^	Y56H + D168V	None^*g*^	None	Y93H/Y	Y93H	Y93H	Y93H
A, relapse, PTW4^*b*^^,^^*d*^	None^*h*^	D168V	D168D/V^*g*^	None	None^*h*^	Y93H	Y93H	Y93H
A, relapse, PTW2*^c,e^*	None^*h*^	D168D/V	None^*g*^	None^*g*^	Y93H	Y93H	Y93H	Y93H
A, relapse, PTW12*^c,e^*	None^*h*^	D168V	None^*g*^	None	Y93H/Y, P58S^*h*^	P58S + Y93H	P58S + Y93H	P58S + Y93H
A, relapse, PTW8*^c,e^*	None^*h*^	D168V^*g*^	None^*g*^	None	Y93H/Y	R30Q + Y93H	R30Q + Y93H	R30Q + Y93H
A, relapse, PTW24*^c,e^*	None^*h*^	D168V	D168V	D168V	A92A/M/T/V, Y93H/Y	Y93H	Y93H	Y93H
B, breakthrough, W12*^b,e^*	None^*h*^	Y56H + D168V	Y56F/H/L/Y, D168D/V^*g*^	None	Y93H	P58S + Y93H	P58P/S, Y93H	Y93H
B, relapse, PTW2^*b*^^,^^*d*^	None^*h*^	Y56H + D168A	D168A/D^*g*^	None	L28M + R30Q, Y93H/Y	L28M + R30Q + Y93H	L28M + R30Q + Y93H	L28M + R30Q + Y93H
C, relapse, PTW8*^b,e^*	None^*h*^	D168D/V	None^*g*^	None	L31M, Y93H/Y	L31M + Y93H	L31M + Y93H	L31M + Y93H
C, breakthrough, W10*^b,e^*	None^*h*^	Y56H/Y, D168A	D168D/H/L/V	D168D/V	Y93H	L31V + Y93H	L31V + Y93H	L31V + Y93H
C, relapse, PTW8*^b,e^*	None^*h*^	D168V	D168D/V	NA	A92A/E, Y93H^*h*^	L31F + A92E	L31F + A92E	NA

a Study M12-536, arm 2 (null and partial responders), received paritaprevir//r (150/100 mg) and ombitasvir (25 mg) QD for 12 weeks; study GIFT-I, arms A and B (noncirrhotics) and arm C (cirrhotics), received paritaprevir/r (150/100 mg) and ombitasvir (25 mg) QD for 12 weeks.

b Treatment experienced with an IFN-containing regimen with or without RBV.

c Treatment naïve.

d IL28B genotype CT.

e IL28B genotype CC.

f Resistance-associated variants were not detected.

g Results by clonal sequencing.

h Results by deep sequencing.

i NA, sample not available; PTW, posttreatment week; W, week; VF, virologic failure; +, linked variants; /, mixture of variants.

**TABLE 7:** *In vitro* activity of paritaprevir and ombitasvir in HCV GT1b-Con1 replicon

Variant(s)	Fold resistance relative to that of the wild type for:		Replication efficiency (%)
	Paritaprevir	Ombitasvir	
NS3			
Y56H	ND		<0.5
D168A	27		69
D168E	4		80
D168V	159		157
Y56H + D168A	700		6
Y56H + D168V	2,472		22
NS5A			
L28M		2	114
R30Q		0.4	44
L31F		10	127
L31M		0.9	119
L31V		8	86
P58S		0.8	80
A92E		0.7	29
Y93H		77	73
Y93S		12	23
L31F + A92E		1,022	36
L28M + Y93H		415	104
R30Q + Y93H		284	60
L31M + Y93H		142	11
L31V + Y93H		12,328	24
P58S + Y93H		1,401	34
L28M + R30Q		0.5	28
L28M + R30Q + Y93H		981	28

a ND, not determined due to the poor replication capacity of the variant.

Variants conferring resistance to paritaprevir in NS3 were not detected at baseline in any of the 13 patients experiencing virologic failure. At the time of failure, 8 patients had D168V, 2 had Y56H + D168A, and 3 had Y56H + D168V. NS3 RAVs persisted in 46.1% (6/13) of the patients through at least posttreatment week 24 and in 17% (2/12) of the patients through posttreatment week 48 by clonal sequencing analysis. In the GT1b replicon, NS3 variants D168A and D168V conferred 27- and 159-fold resistance to paritaprevir, respectively, and the addition of Y56H to one of these variants increased resistance by an additional 15- to 26-fold.

In NS5A, Y93H alone or in combination with L31M, P58S, A92E, or L28M + R30Q was detected in 10 patients at baseline. At the time of failure, 1 patient had L31F + A92E, 5 had Y93H, and 7 had Y93H in combination with L28M, R30Q, L31M/V, and/or P58S. In patients with available data, RAVs in NS5A remained detectable through posttreatment week 48. In the HCV GT1b replicon, NS5A variants L31F and Y93H confer 10- and 77-fold resistance, respectively, to ombitasvir. Variant L28M, R30Q, L31M, or A92E did not confer resistance, but double variants of A92E in combination with L31F or of L28M, R30Q, L31M/V, or P58S in combination with Y93H conferred an additional 2- to 160-fold resistance to ombitasvir.

HCV GT1b replicons containing amino acid variants in NS3 had replication efficiencies varying between < 0.5% and 157%, and variants in NS5A had replication efficiencies varying between 11% and 127% relative to that of the wild-type replicon. In clinical studies, NS3 RAVs, including those with high replication efficiencies, did not persist through posttreatment week 48, while NS5A RAVs, including those with low *in vitro* replication efficiencies, persisted through posttreatment week 48. The lack of correlation between replication efficiencies observed *in vitro* and the persistence of RAVs in patients treated with paritaprevir and ombitasvir who experienced virologic failure indicates that there are limitations in the use of the replicon assay for assessing *in vivo* viral fitness.

## DISCUSSION

Phase 2 study M12-536 and phase 3 study GIFT-I assessed the 2D regimen containing paritaprevir/r and ombitasvir in Japanese HCV GT1b-infected patients. High SVR rates were observed with an overall virologic failure rate of 3% (13/436) (13, 14).

Baseline sequence analysis of the NS3 and NS5A genes was conducted to evaluate geographic differences in the distribution of polymorphisms at amino acid positions that are important for the activity of NS3 protease or NS5A inhibitors. The pattern generally was similar for both Japanese and western patient populations. However, there were some differences in the geographic distribution of specific NS3 and NS5A polymorphisms. Q80L and S122G in NS3 and L28M, R30Q, and Y93H in NS5A were detected in a significantly higher proportion of Japanese patients than western patients (predominantly from the United States and Europe). Similar differences by geographic region were observed previously in the baseline HCV GT1 sequence analysis in the AVIATOR study, where all GT1a sequences encoding M28V in NS5A were from the United States, while GT1b sequences encoding C316N and S556G in NS5B were predominant in Europe (25). Geographic differences in the prevalence of Q80K in NS3 in GT1a are well documented, with higher prevalence in the United States than in Europe (34). A longitudinal phylogenetic analysis (by geographic region and time of sample collection) of HCV GT1a sequences by McCloskey et al. indicated that the majority of the NS3 Q80K-carrying sequences (96%) have descended from a single substitution event that occurred over 50 years ago in the United States, perhaps accounting for the higher prevalence of the variant in this region (10). As HCV infection was prevalent in Japan before North America and Europe, viral evolution and transmission may have been confined to a restricted geographic region for a period of time, leading to regionally specific sequence variability. However, longitudinal phylogenetic analysis of sequences will be required to understand these differences.

Baseline paritaprevir resistance-conferring variants in NS3 were rarely observed in Japanese or western patients. L31F and Y93H/S in NS5A were the only variants that conferred resistance to ombitasvir, and they were detected in 0.5% and 12.5% of the Japanese patients, respectively, by population sequencing. The baseline prevalence of NS3 and NS5A polymorphisms also has been presented by Manns et al. from a non-Japanese multinational clinical trial of asunaprevir and daclatasvir conducted in 18 countries, including the Asian countries South Korea and Taiwan, with a data set size of >600 patients (29). In this study, D168E in NS3 was detected in 0.6% of the patients and L31F/I/M/V and Y93H/S in NS5A were detected in 4.5% and 8.0% of the patients, respectively, by population sequencing, similar to our observations in the western patient population (29). Baseline analysis in the GIFT-I study also was conducted by deep sequencing. Resistance-conferring variants in NS3 were not detected using a detection threshold of 1%, while L31F and Y93H in NS5A were detected at a prevalence of 1% and 21%, respectively. Comparison of population and deep-sequencing data indicated that the detection limit of population sequencing was approximately 15%.

Polymorphisms in NS3 and NS5A, with the exception of Y93H in NS5A, had no impact on treatment outcome in studies M12-536 and GIFT-I. In study M12-536, the prevalence of Y93H in NS5A at baseline was 5.6% by population sequencing, and all 4 patients with this variant at baseline achieved SVR_24_. In GIFT-I, the SVR_24_ rate in patients with Y93H present in ≥1% of their viral population was 85% (63/74), whereas the SVR_24_ rate was >99% in patients without Y93H (<1% by deep sequencing). The most informative data subset for the prediction of treatment outcome was a detection threshold of 40% for Y93H. In the GIFT-I study, 9% of the noncirrhotic and cirrhotic patients met this criteria, and the SVR_24_ rate in this subset was 76% (25/33) compared to an SVR_24_ rate of >98% (314/318) in patients with <40% prevalence of Y93H (including those with wild-type Y93). Although the presence of Y93H was associated with a lower SVR_24_ rate, the majority of the patients with this variant at baseline achieved SVR_24_, suggesting that factors other than the presence of Y93H in NS5A impact treatment outcome. Population sequencing-based tests (detection threshold of >15 to 20%) as well as more quantitative tests to evaluate the presence of Y93H in NS5A at baseline are available to clinicians in Japan. The results from this study may be informative for physicians making decisions regarding treatment in GT1b-infected patients who have Y93H in NS5A at baseline.

Among the 13 virologic failures in the phase 2 and 3 studies, RAVs in NS3 as well as NS5A were observed in all 13 patients after failure. D168A/V alone or in combination with Y56H in NS3 and L31F plus A92E or Y93H alone or in combination with L28M, R30Q, L31M/V, and/or P58S in NS5A were detected at the time of failure. Treatment-emergent RAVs in NS3 declined over time, whereas RAVs in NS5A remained detectable through posttreatment week 48.

A limitation of this study is that the results are pertinent only to the use of the 2D regimen in Japanese HCV GT1b-infected patients. In the United States, Europe, and other countries worldwide, where a greater proportion of people are infected with GT1a than that observed in Japan, the 2D regimen in combination with dasabuvir (a nonnucleoside NS5B inhibitor), with or without RBV, is approved for the treatment of HCV GT1-infected cirrhotic and noncirrhotic patients (20–27). Therefore, the impact of RAVs on treatment outcome described in this study of the 2D regimen is not applicable to the western patient population.

In summary, Japanese GT1b-infected patients treated with paritaprevir/r and ombitasvir achieved high SVR rates. Certain NS3 and NS5A polymorphisms were detected at a higher prevalence in the Japanese population than in the western population. The impact of baseline RAVs on treatment outcome was limited to Y93H in NS5A; however, a majority of patients with this variant achieved SVR.
